# How the Ketogenic Diet Shapes the Microbiome to Influence Cancer Immunotherapy Outcomes: An Exploration of Clinical Trials and Their Results

**DOI:** 10.1080/01635581.2026.2658807

**Published:** 2026-05-04

**Authors:** Jonah Bukovac, Marium Husain, Teryn Sapper, Angela O’Dell, Marisa Bittoni, Helena Gastier, Ashwini Chebbi, Victoria Hockenhull, Zachary Dohar, Jennifer Moon, Fred Tabung, Claire Verschraegen, Richard Wu, Kari Kendra, Yuanquan Yang, Jeff Volek, Daniel Spakowicz

**Affiliations:** a Pelotonia institute for Immuno-Oncology, The Ohio State University Comprehensive Cancer Center, Columbus, OH, USA; b Division of Medical Oncology, Department of Internal Medicine, The Ohio State University Comprehensive Cancer Center, Columbus, OH, USA; c Department of Human Sciences, The Ohio State University, Columbus, OH, USA; d Comprehensive Cancer Center, The Ohio State University, Columbus, OH, USA; e Center of Microbiome Science, The Ohio State University, Columbus, OH, USA

## Abstract

Ketogenic dietary interventions (KDIs) are increasingly explored as adjuncts in oncology due to their metabolic and immunomodulatory effects. One mechanism by which KDIs are expected to modulate the immune system is by altering the gut microbiome, which has been shown to affect treatment outcomes, particularly in the context of immunotherapies. This review synthesized findings from 43 clinical trials to evaluate the current landscape of KDIs in cancer care, with a focus on the gut microbiota and immunotherapy. Although 47% of identified trials are completed, none have yet published results combining KDIs with immunotherapy. Since 2020, however, there has been a significant increase in ongoing studies investigating this combination and incorporating microbiome endpoints. While KDIs may help shape an immunotherapy-permissive environment, further clinical evaluation is necessary to determine the full extent of KDIs on the microbiome. Future research should prioritize longitudinal microbiome profiling and standardized adherence reporting to clarify the therapeutic potential of KDIs as a metabolic adjuvant to immune checkpoint inhibitors.

## Introduction

Cancer remains the second leading cause of death worldwide (1), driving the ongoing search for more effective and efficient treatment options. In 2025, there are projected to be 618,120 deaths from cancer in the United States alone (2). Among the most significant advancements in oncology over the past decade is the massive increase in the use of immunotherapy—specifically immune checkpoint inhibitors (ICIs)—across a wide array of malignancies (3,4). By releasing inhibitory immune checkpoints, immunotherapy enhances the cytotoxic function of endogenous antitumor T cells and has produced durable clinical responses for many cancer types, redefining the standard of care for various tumors (5–8). However, most tumors do not exhibit a strong, durable response, underscoring the urgent need for strategies to enhance the efficacy of these treatments (9).

Crucially, the gut microbiome has emerged as a primary mediator of this immunotherapy response (10–12). Microbial species produce metabolites that enter host circulation and modulate both local and systemic immune activation by acting on immune cells and their receptors (13,14). For example, certain organisms produce short-chain fatty acids (SCFAs) that enhance the response to immune checkpoint blockade by expanding T regulatory and effector cytotoxic CD8^+^ T cell populations (15–17). Inosine, produced by organisms such as *Bifidobacterium pseudolongum*, activates CD8^+^ T cells through adenosine receptor binding (18), while *Enterococcus* species release peptidoglycan fragments or flagellin that stimulate the NOD2 and TLR5 pathways to boost CD8^+^ infiltration (19). As the link between microbial composition and immunotherapy outcomes becomes more established, identifying ways to therapeutically modulate this axis has become a clinical priority.

Dietary modifications are an attractive, noninvasive way to intervene in the microbiome (20). Multiple controlled feeding studies have shown that even short-term dietary changes can rapidly shift microbial community structure and metabolic outputs, highlighting diet’s potential as a targeted strategy to modulate the microbiome for health benefits (21). Diet influences treatment response through both direct host effects and indirect microbial effects. Directly, food-derived nutrients in the tumor microenvironment can regulate metabolic reactions in both tumor and immune cells, which, in turn, modulate T-cell metabolism and alter anti-tumor effects (22–25). Nutrient competition between tumors and T cells, particularly for glucose, can regulate the tumor microenvironment and directly impact cancer progression (26). This competitive nutrient landscape is driven in part by the Warburg effect, in which tumor cells engage in aerobic glycolysis in the presence of oxygen, leading to excessive glucose consumption and impairing the mTOR signaling, glycolytic activation, and cytokine production of infiltrating T cells, thereby weakening anti-tumor immunity (27–29).

Notably, the ketogenic diet (KD)—a high-fat, low-carbohydrate intervention—is an underappreciated tool in this regard. Although the KD has been studied for decades, its emphasis has focused almost exclusively on host metabolic effects, including the ability to mimic fasting and accelerate lipolysis. These effects are often characterized by reduced insulin levels and increased fatty acid release, which undergo β-oxidation and ketogenesis in the liver (30–32), leading to the physiological elevation of the two main circulating ketone bodies, β-hydroxybutyrate (BHB) and acetoacetate (33,34). In cancer, these ketone bodies have demonstrated broad-spectrum metabolic and signaling functions in tumors, including modulating immune cell metabolism and enhancing anti-tumor responses by restoring T-cell function and inhibiting cancer cell proliferation (35–37). Furthermore, by shifting host metabolism away from glucose, the KD may mitigate nutrient competition between glycolytic tumors and T cells, thereby overcoming a known barrier to immune checkpoint inhibitor efficacy (26,38). Additionally, ketone bodies such as BHB function as epigenetic modifiers that can directly upregulate effector pathways in cytotoxic T cells, potentially acting as sensitizers for immunotherapy (36). The potential for KDs to shape the microbiome into an “immunotherapy-permissive” state is a much newer frontier that bridges metabolic therapy with immune-oncology.

Despite this potential, there remains a significant gap between mechanistic theory and clinical application. While early trials focused on KD safety and host effects, a new generation of clinical trials is beginning to incorporate diverse endpoints and evaluate its potential as an adjunct cancer therapy. The effects of KD on the microbiome and its anti-tumorigenic properties remain critical questions for clinical translation. This review examines the current clinical evidence on ketogenic dietary interventions in cancer patients, with a focus on the state of the gut microbiota and the implementation of immunotherapy endpoints in studies. We also explore the different dietary forms, ketosis biomarkers, and adherence across ketogenic dietary interventions in oncology. Through these synthesized findings, this review highlights the current progress in human trials and offers a framework for an intersecting field that holds potential to shape future cancer therapeutic strategies.

## Methods

### Search Terms and Eligibility Criteria

The literature search was conducted in May 2025. A primary search was conducted using ClinicalTrials.gov as the database, while a secondary search was conducted using PubMed, Google Scholar, and reference lists. From the ClinicalTrials.gov database, an Expert Search was performed with a combination of the following parameters as search terms: “ketogenic diet,” “low carbohydrate diet,” “Atkin’s diet,” “cancer,” “treatment,” “gut microbiota,” “immunotherapy,” “chemotherapy,” “radiation therapy,” and “chemoradiation.” We included RCTs, single-group assignments, parallel-group assignments, and crossover trials. Animal studies, retrospective studies, case reports, case series, and cohort studies were omitted. Studies that were not in English were also excluded.

### Data Retrieval and Classification

Trials were classified as (1) Completed or (2) In-progress, Terminated, and Withdrawn. All retrieved records were screened for relevance and consistency by a single reviewer (JB). Both primary and secondary literature searches were conducted using predefined search parameters. Eligibility was determined based on established criteria: (1) interventional study design, (2) inclusion of human subjects, (3) relevance to ketogenic diet and cancer treatment, and (4) availability of treatment details, outcome data, and adherence information (for completed trials). Consistent application of the inclusion and exclusion criteria was maintained throughout the screening process to minimize selection bias by the reviewer. We retrieved information on treatment, adherence, cancer type, results, phase, initiation date, study design, intervention, cancer type, treatment, outcome measures, sample size, and adherence. Treatment was defined as a concurrent regimen with KD, regardless of the line of therapy (i.e., first-line vs. second-line ICI treatment). Sample size was determined based on the number of patients at baseline and/or enrolled in the KD intervention in each respective trial.

### Data Visualization

Trial data were visualized using the ggplot2 package in R 4.1.1 (39).

## Results

A comprehensive search for cancer clinical trials involving a ketogenic dietary intervention yielded 43 trials. Based on our predefined criteria, 20 (47%) trials were identified as completed, 13 (30%) trials were classified as in-progress, and 10 (23%) were identified as terminated or withdrawn. For the purposes of this review, two trials (NCT01865162 and NCT02302235) were treated as a single completed trial based on their joint reporting in a single published manuscript. We categorized one trial (NCT04631445) as completed despite the publication of only preliminary findings (40) at the time of this review.

**Figure 1.**
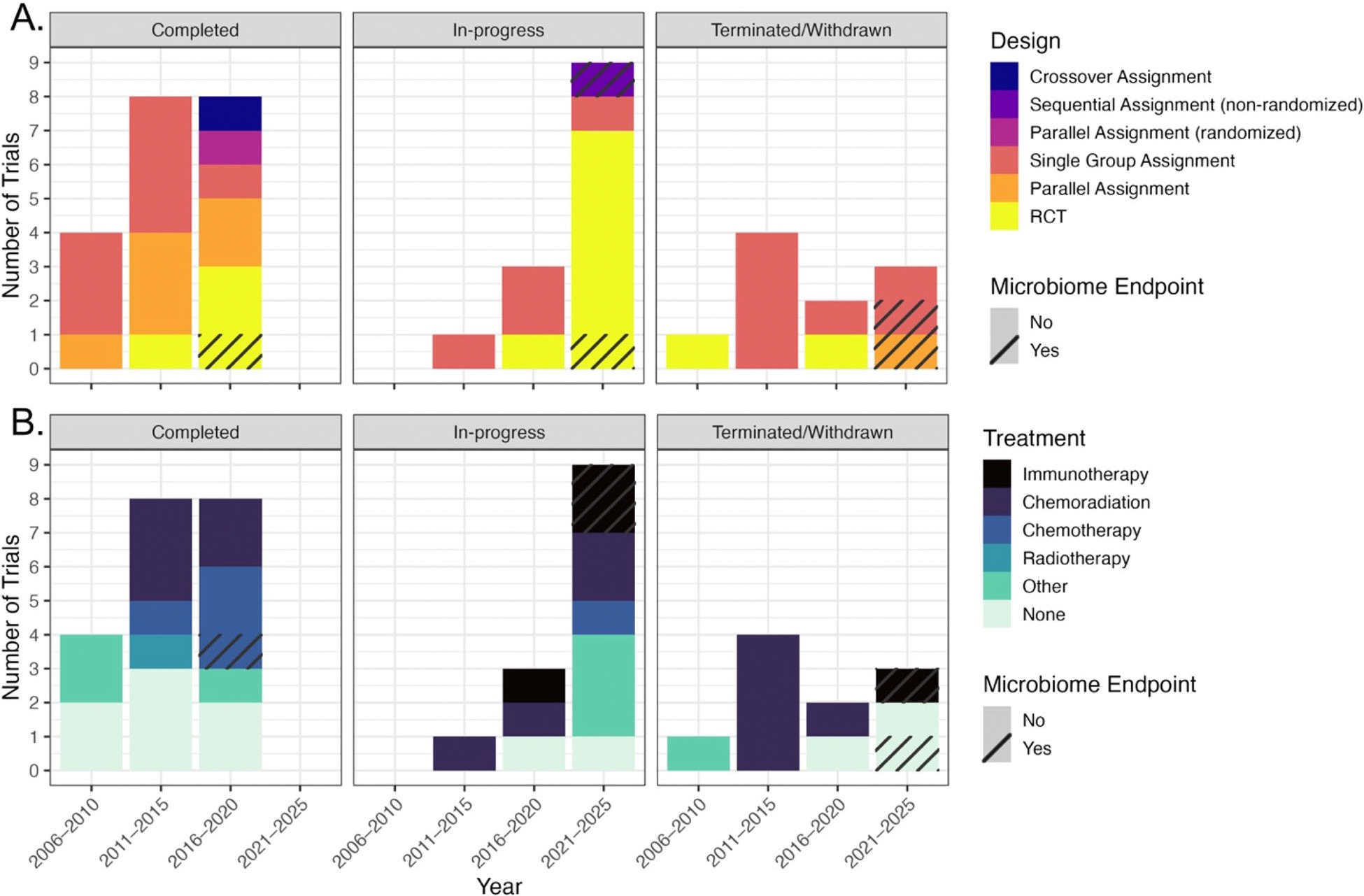
The number of trials by time-period for completed, in-progress, and terminated or withdrawn KD intervention and cancer studies. The number of trials in each period is subdivided by design (A) or concurrent treatment (B).

Across completed, in-progress, and terminated/withdrawn trials, there has been a noticeable expansion in the diversity of concurrent treatment arms evaluated alongside KD intervention (Figure 1). Notably, to date, no completed KD trial has incorporated immunotherapy as a concurrent treatment modality. However, there is a notable increase in in-progress trials investigating the effects of KD intervention in combination with concurrent immunotherapy treatment (Figure 1).

Specifically, one trial (NCT04316520) initiated in 2020 planned to evaluate the tolerance of a 2:1 KD in metastatic renal cell carcinoma (mRCC) patients alongside concurrent pazopanib, sunitinib, pembrolizumab/axitinib or nivolumab/ipilimumab (for first line), nivolumab, cabozantinib, axitinib, sorafenib, TKI anti-VEGF (for second and third line of treatment). The study plans to implement a 1-year KD intervention, while also examining adherence, progression-free survival (PFS) at 2 years, and overall survival (OS) at 2 years.

Additionally, in 2025, two trials examining KD intervention alongside concurrent immunotherapy were initiated. One study (NCT06391099) aims to assess the feasibility and adherence of a 24-week, 2:1 KD, while evaluating the improvement of immunotherapy response in mRCC and metastatic melanoma (MM) patients receiving concurrent combination nivolumab and ipilimumab or single-agent ipilimumab, nivolumab, or pembrolizumab. The other trial (NCT06896552) intends to explore the impact of a 10-week intermittent KD on immune function and treatment outcomes in patients with MM, metastatic cutaneous squamous cell carcinoma, and mRCC receiving concurrent treatment with first-line combination nivolumab and ipilimumab/relatimab, or single-agent ipilimumab, nivolumab, pembrolizumab, or cemiplimab.

In contrast, one trial (NCT05119010) studied the objective response rate of concurrent nivolumab and ipilimumab in mRCC patients receiving either oral BHB supplementation, continuous, or discontinuous KD for 3 months. The trial also planned to evaluate the safety of immunotherapy in relation to KD intervention, assess quality of life (QoL), and measure OS and PFS. However, the study was ultimately terminated due to recruitment challenges and issues with biological sample collection.

Along with the increasing interest in immunotherapy- based concurrent treatment regimens with KD intervention, a subset of trials over time has incorporated or established defined microbiome-associated endpoints. A total of five trials retrieved from our search have established microbiome endpoints, four of which were initiated from 2021 up to the time of this review.

Two of the trials that have explicitly established microbiome endpoints are currently in progress and were initiated in 2025. One trial (NCT06896552) aims to investigate the efficacy of a structured KD in enhancing immune response and treatment outcomes when combined with immune checkpoint inhibitors (ICIs) in patients with advanced melanoma, cSCC, or RCC. The study team plans to analyze fecal microbiome samples at baseline and at the end of the 10-week treatment, which includes, but is not limited to, 16S analysis. Another in-progress trial (NCT06391099) aims to evaluate the safety and feasibility of KD in patients with metastatic RCC and melanoma who are receiving ICIs. Additionally, the study plans to collect biospecimen samples at three time points for microbiome analysis and mechanistic studies. The study team plans to investigate if the microbiome (binary, high versus low diversity) mediates the relationship between time in ketosis and tumor size over the course of treatment.

In contrast, two trials with explicit microbiome endpoints were terminated or withdrawn in 2023. One terminated study (NCT0511901) planned to evaluate whether KD or BHB supplementation could improve response rates to ICIs in patients with metastatic RCC. In the context of the microbiome, the study team planned to explore whether the induction of ketosis and ketone metabolism shifts and modulates the gut microbiome. Another study (NCT04231734) was withdrawn and had planned to explore the feasibility of KD in patients with untreated low-tumor-burden mantle cell lymphoma. The study aimed to evaluate the effects of KD on the gut microbiome by identifying shifts in gut bacteria using DNA analysis of stool samples at 12 weeks.

Noticeably, only one completed study (NCT04631445) from our search was identified to establish definitive microbiome endpoints. While further results are pending, preliminary findings from the trial suggest that the study team intends to analyze the gut microbiome as a secondary endpoint. Furthermore, the study plans to evaluate the safety and feasibility of an implemented medically supervised ketogenic diet (MSKD) in patients with metastatic pancreatic cancer receiving triplet chemotherapy.

### Completed Ketogenic Diet Intervention Cancer Clinical Trials

While these completed trials did not specifically target immunotherapy outcomes, evaluating their adherence rates, safety profiles, and metabolic efficacy is a prerequisite for determining the feasibility of KD as an adjuvant to complex immunotherapy regimens. Across the 20 completed trials, a total of 704 patients enrolled or participated (Tables 1–3). Of these, 378 patients (54%) received a ketogenic diet or a variation of it (e.g., MAD, AD, MSKD, MKD, IID, WFKD, see Box 1), while the remaining 326 patients (46%) served as controls or were assigned to a non-KD intervention. Overall, 540 patients (77%) completed their trials through to the final endpoint. Among patients on KD, 266 (70%) completed the intervention through to the final endpoint. Of those who discontinued or were withdrawn from KD interventions, 29 patients were reported to have done so due to either inability to comply or the dietary burden associated with the KD.

Our search identified four RCTs investigating KD interventions in cancer patients that were conducted and fully completed between 2006 and May 2025. Notably, three of these RCTs have published complete findings, while one has only reported preliminary results as of May 2025. Among the three RCTs with published results, a total of 193 patients were randomized, with 97 (50%) assigned to the KD intervention. Of those in the KD group, 71 (73%) completed their assigned diet until the final endpoint. Overall, 141 (73%) of all enrolled patients completed their randomized regimen. The mean duration of the three trials was 13.33 weeks. All RCTs used clear methods to measure dietary adherence, although the specific biomarkers and diet forms varied across trials.

### Ketogenic Diet Interventions and Adherence

With respect to adherence, we chose to highlight the trials that utilize randomized controlled designs. These were selected as they represent high-level clinical evidence that utilize robust study designs to evaluate patient adherence and metabolic outcomes.

In one trial (NCT03171506), 37 patients with ovarian or endometrial cancer were randomized to a macronutrient KD ratio of 70% fat, 25% protein, and 5% carbohydrates for 12 weeks (Table 4). Of these, seven patients received concurrent chemotherapy treatment. Adherence was assessed on urinary ketone levels, but levels were not explicitly established. Four patients withdrew from the KD arm due to inability to tolerate or adhere to the diet. Additionally, the KD group (0.91 ± 0.16 mmol/L) achieved significantly higher serum BHB levels than the control group (0.25 ± 0.04 mmol/L), (*p* < 0.001) (41) (Table 4).

Another trial (IRCT20171105037259N2) used a 55% fat, 19% protein, and 6% carbohydrate macronutrient ratio, with 20% of energy derived from medium-chain triglycerides (MCTs) (Table 4). In total, 40 breast cancer patients were randomized to the KD arm and received concurrent chemotherapy treatment with KD for 12 weeks. Ketosis was defined as a serum BHB concentration of greater than 0.3 mmol/L, while dietary adherence was based on BHB levels and dietary intake. 89% of patients in the KD arm achieved ketosis and were considered adherent. Two patients discontinued the study due to their inability to comply with the diet (Table 4) (42).

In a third trial, a modified ketogenic diet (MKD) was administered with the macronutrient breakdown of 75% fat, 15% protein, 10% carbohydrate in 20 advanced cancer patients for 16 weeks, while receiving concurrent chemotherapy or radiation therapy (Table 4). Three-day food diaries were collected at weeks 6 and 16 to assess dietary adherence, along with the defined ketosis parameters of urinary ketones greater than 0.5 mmol/L. Explicit urinary ketone levels were not reported, but most patients took ~2 weeks to achieve and maintain ketosis. Additionally, there were no patient dropouts related to dietary adherence (Table 4) (43).

A fourth RCT (NCT04631445) reported initial findings from the implementation of an unspecified MSKD on 16 randomly assigned pancreatic cancer patients, concurrent with chemotherapy (Table 5). Ketosis was defined as BHB levels between 0.5 and 3.0 mmol/L. In total, 15 patients achieved nutritional ketosis, with a mean BHB level of 0.57 mmol/L (95% CI, 0.40–0.73). Additionally, KD patients spent a median of 39.4% of study duration in ketosis (range 0–95.8%). Further results and analysis are pending at the time of this review (Table 5) (40).

### Tumor Response and Survival Outcomes of Ketogenic Diets in Cancer Therapy

From our search, we identified several studies that evaluated efficacy measures of KD through tumor progression and survival rates. In a 12-week RCT involving patients with locally advanced metastatic breast cancer, KD induced a significant systemic shift characterized by suppression of the pro-inflammatory cytokine TNF-α and an increase in the anti-tumorigenic cytokine IL-10, along with significantly reduced fasting insulin and IGF-1 levels (42). Participants in the KD arm demonstrated a four-fold greater reduction in tumor size compared to controls (27 mm vs. 6 mm, *p* = 0.01), along with significant pathological downstaging of the TNM (Tumor, Node, Metastasis) index (*p* < 0.01) (Table 4) (42). Notably, these results were most profound in patients with locally advanced disease and did not extend significantly to the metastatic subgroup. Similar efficacy was observed in non-metastatic rectal cancer patients undergoing chemoradiation, with the KD group achieving higher tumor regression grades and a significantly higher rate of near-complete tumor response (43%) than the control group (15%) (*p* = 0.116) (Table 6) (44). A larger intention-to-treat analysis in the KD group (50%) compared to the control group (14%) (*p* = 0.018) was noted, but did not reach statistical significance (44).

In glioblastoma cohorts, outcomes varied and revealed trends associated with metabolic adherence. One trial involving glioblastoma patients receiving MKD or MCTKD reported a median time to progression of 14.4 weeks (SE: 14.6, 95% CI: 0–42.9 week) and a median overall survival of 67.3 weeks (SE: 6.2, 95% CI: 55–79.6 weeks) (Table 7) (45). In a trial involving patients with progressing glioblastoma, those who achieved stable ketosis showed a trend toward longer PFS compared to those who did not (*p* = 0.069) (Table 8) (46). Furthermore, in a pilot study of untreated prostate cancer patients, 50% of KD patients exhibited tumor remission or downgrading on re-biopsy, but notably did not show any significant shifts in inflammatory biomarkers or PSA (*p* > 0.05) (Table 5) (47). Notably, in a pilot study of 10 patients with advanced cancer, those who achieved stable disease or partial remission exhibited a 17-fold increase in relative ketosis, compared to only a 5-fold increase in those with progressive disease (*p* = 0.018) (Table 7) (48). Both groups experienced a similar 4% reduction in body weight and ~35% caloric deficit, but only the group with higher ketone levels achieved stable disease, with decreases in fasting insulin of 75% and 90% in responders (48). Although these studies did not explicitly measure immune endpoints, the observed tumor regression and reduction in insulin/IGF-1 levels suggest the creation of a metabolic environment that may impact immune surveillance, an important factor for the success of future immunotherapy-combined trials.

### In-Progress Ketogenic Diet Intervention Cancer Clinical Trials

In the context of ongoing clinical trials, there is a growing interest in ketogenic diets (KDs) as additional treatments in cancer therapy, especially when combined with immunotherapy and chemoradiation (Tables 9–11). There has also been a broader range of cancer types studied, including melanoma, renal cell carcinoma, glioblastoma, breast, rectal, and endometrial cancers. Additionally, protocols differ widely in macronutrient makeup, implementation methods (e.g., continuous vs.. cyclic), and duration, highlighting the exploratory nature of this area. Several trials are examining not only clinical outcomes like tumor response and survival but also mechanistic links such as metabolic biomarkers, immune modulation, and gut microbiome diversity. Notably, multiple studies focus on feasibility, safety, and patient adherence, which are vital for incorporating dietary interventions into standard oncology practice. There has also been increased interest in RCTs, with seven currently ongoing.

### Terminated or Withdrawn Ketogenic Diet Intervention Cancer Clinical Trials

Regarding the identified terminated and withdrawn trials, most used single-arm designs, with only two structured as RCTs (Tables 12–14). The main reasons for early termination included low patient recruitment, poor adherence to strict dietary protocols, funding issues, and competition for study enrollment. These challenges were observed across various cancers, such as glioblastoma, pancreatic, lung, prostate, and pediatric brain tumors, highlighting common feasibility issues when trying to incorporate complex nutritional strategies into standard cancer treatments. Notably, one trial (NCT05119010) involving immunotherapy and microbiome approaches was terminated due to recruitment problems caused by competition with another microbiome trial, patient refusal, and poor tolerance of oral DPD (Table 12).

## Discussion

### Emerging Role of Ketogenic Diets in Cancer Therapy

This review highlights the dynamic and evolving landscape of ketogenic diet exploration in oncology clinical trials. Across 43 identified clinical trials, 20 trials (47%) were completed, while 23 (53%) remain in progress, and 10 (23%) were terminated or withdrawn. Among the completed trials, 704 patients were enrolled, and 378 (54%) were assigned to KD, with 266 (70%) completing the study through its final endpoint. While no completed trials to date have combined KD with immunotherapy, a growing number of studies since 2020 have actively explored this approach. Correspondingly, there has been an increase in the implementation of microbiome endpoints in ongoing ketogenic cancer trials since 2021, with pending results from a study initiated in 2020 and completed at the time of this review. This reflects the growing interest in the potential immunomodulatory role of the gut microbiome in the efficacy of KD as an adjuvant cancer therapy. Notably, of the two in progress trials with microbiome endpoints, both plan to incorporate immunotherapy as a concurrent treatment with KD. In the context of RCTs, while only four have been completed as of May 2025, seven RCTs have been initiated since 2020, highlighting the interest in exploring KD in controlled environments. This progression from single-arm metabolic studies to randomized immunotherapy-microbiome designs illustrates the maturation of the field. By synthesizing the broader landscape of 43 trials, we have identified key feasibility barriers—such as recruitment challenges and protocol rigidity—that must be addressed to optimize the success of current and future trials focusing on immunotherapy and microbiome modulation. Collectively, these findings underscore an emerging clinical interest in KD as a complementary cancer therapy, with a particular emphasis on its combination with immunotherapy and modulation of the microbiome.

### Synthesis of Clinical Findings

An integrated analysis of the tabulated findings reveals three distinct trends that define the current trajectory of ketogenic therapies in oncology. First, a cross-comparison of completed (Tables 1–8) and terminated studies (Tables 12–14) illustrates a critical tradeoff between metabolic rigor and trial feasibility. While classic protocols (e.g., 4:1 ratios) generally drove higher ketone biomarkers (Tables 4—8), they were disproportionately represented in terminated trials in which dietary burden and inability to comply were cited as primary drivers of withdrawal (Tables 12–14). Conversely, trials utilizing MAD or MKD (Tables 1–3) demonstrated higher completion rates but often reported variable induction of ketosis. Taken together, these findings suggest that future trial designs may need to balance maximal metabolic suppression (classic KD) against patient retention (MAD/MKD), as these objectives appear inversely associated in the current clinical landscape. However, this synthesis should be interpreted cautiously given the heterogeneity of the evidence base, including small sample sizes, inconsistent ketone metrics and reporting timepoints, and variability in cancer types and levels of dietary supervision across studies.

Additionally, the progression from completed trials (Tables 1–3) to in-progress trials (Tables 9—11) highlights a shift in experimental intent. Whereas completed trials were predominantly single-arm pilot studies focused on safety and feasibility, the current landscape (Tables 9—11) is increasingly characterized by randomized controlled trials designed to evaluate efficacy endpoints. Notably, this shift is marked by the emergence of combinatorial designs, in which KDs are no longer tested solely as metabolic monotherapies but as adjuvants to immunotherapy (Table 9). This evolution reflects the growing hypothesis that KD interventions may function not only as metabolic inhibitors but also as immune sensitizers.

Evidently, the clinical landscape of KD interventions in oncology is rapidly evolving, particularly concerning their role as a metabolic adjuvant to immunotherapy. While current completed trials provide a foundation for safety and metabolic adherence, the lack of published microbiome data in cancer-specific KD cohorts remains a significant knowledge gap. However, the increasing number of in-progress trials (Figure 1) suggests that we are entering a phase where the clinical relevance of these microbial shifts will soon be evaluated within oncological populations.

### Immunotherapy and Ketogenic Dietary Cancer Interventions

Recent animal models have established a strong foundation for preclinical evidence on the interactions between the ketogenic diet and immunotherapy. Notably, a 2020 study found that intermittent oral 3-hydroxybutyrate supplementation restored the therapeutic response to anti-PD-1 treatment in mice that had previously failed to suppress tumor growth (35). Conversely, anti-tumor effects were abolished when ketogenesis was disrupted by sucrose supplementation or an antagonist that binds to the 3-hydroxybutyrate receptor. Additionally, a 2024 study showed that exposure to ketone bodies through an ad libitum 2:1 KD intervention was associated with reduced tumor cell growth and proliferation, along with an improved response to anti-PD-L1 mAb treatment in mice with renal cell carcinoma (49). In a CT26 mouse model, KD increased intratumoral infiltration of CD8+ cytotoxic T cells and Th1-polarized CD4+ T cells (50). Moreover, KD downregulated PD-L1 and CTLA-4 expression, highlighting its potential to modulate multiple immune checkpoints. Mechanistic studies suggest that ketogenic diets create a metabolic environment that may be unfavorable for tumor cells while supporting T cell effector function. For example, recent evidence indicates that ketone bodies, such as BHB, can enhance CD8+ T cell bioenergetics and effector gene expression, counteracting features of T cell exhaustion and potentially improving responses to PD-1 blockade (35,36,51). Furthermore, reducing systemic insulin, such as through a KD, can downregulate the PI3K/Akt/mTOR pathway in tumor cells, a pathway that has been implicated in promoting immune evasion, providing a mechanistic rationale for combining KD with immunotherapy (52).

Overall, preclinical and mechanistic evidence suggests promising potential and rationale for KD as a metabolic adjuvant to ICIs, particularly when combined with PD-1/PD-L1 or CTLA-4 blockade. These recent findings contribute to growing interest in exploring the immunomodulatory effects of KD in cancer patients, and three trials initiated since 2020 are currently examining this. These studies (NCT06391099, NCT06896552, NCT04316520) aim to address a key gap in translational oncology by assessing the feasibility, safety, and outcomes of ICIs in patients with advanced cancer. Specifically, they intend to evaluate the immune response and treatment efficacy of the ketogenic diet in patients with advanced melanoma, cSCC, and RCC. The lack of completed trials underscores the limited clinical data available and the urgent need for well-designed studies to determine the effectiveness, outcomes, and mechanisms of the ketogenic diet as an adjuvant to immunotherapy.

### Gut Microbiome as a Mediator of Ketogenic Diet Efficacy

While the direct metabolic effects on T cells are compelling, the gut microbiome represents a critical secondary pathway through which KD may influence the clinical efficacy on the immunotherapies discussed above. Recently, the gut microbiome has become a key area of interest in investigating tumor outcomes and the efficacy of KD as an adjuvant treatment in cancer patients. In non-cancer patients and mice, KD has been shown to induce significant shifts in the gut microbiome. Notably, KD has been shown to increase abundances in *Bacteroidetes* (53–56), while significantly decreasing abundances in *Bifidobacteria* (53,56). Notably, there have been conflicting results in the influence of KD on the bacterial abundance of *A. muciniphila*, as some studies have reported increases (57,58), while others have reported a decrease in bacterial abundance (59,60). In the absence of published cancer-specific results, the potential for KD to shape an “immunotherapy-permissive” microbiome remains a strong mechanistic hypothesis. In cancer patients, understanding the interactions between diet and gut microbiota is crucial, as they have the potential to modulate primary and secondary lymphoid tissues, which in turn impact tumor immunosurveillance (61). Additionally, it is well known that ICI efficacy and immune response are impacted and influenced by the composition of the gut microbiome (10,62–69).

Biologically, these shifts represent a broad restructuring of the gut ecosystem away from saccharolytic species that rely on carbohydrate fermentation. This transition carries significant clinical implications: the depletion of *Bifidobacterium* and associated drop in SCFAs observed in standard KD formulations (70) suggests a decline in fiber fermentation, which could potentially compromise gut barrier integrity and immune regulation (71–73). This functional deficit is further complicated by the observed expansion of the *Firmicutes* phylum (70). While this phylum typically harbors butyrate-producing genera essential for tumor suppression and barrier maintenance (74–76), and has been linked to improved anti-PD-1 efficacy through CD8+ T cell activation (77), the simultaneous reduction in SCFA output in the study suggests a functional decoupling. This may imply that without adequate fiber substrate, the KD-expanded *Firmicutes* may fail to provide the anti-inflammatory metabolites necessary to protect the gut mucosa during cytotoxic cancer therapies. Consequently, these alterations may impair anti-tumor responsiveness, given the established roles of microbiome-derived SCFAs and gut barrier integrity in shaping system immune regulation and responses to immune checkpoint inhibitors (71–73). However, this effect appears dependent on specific dietary composition; for example, in one study Mediterranean-style KDs have been shown to preserve butyrate production (78) or have no significant difference in production (53), suggesting that fiber source and fat quality can mitigate potential dysbiosis.

Beyond shifts in dominant phyla, the enrichment of the genus *Akkermansia* (notably *A. muciniphila*) in human cohorts (70,78) represents a finding of high clinical significance in oncology. As a mucin-degrading bacteria, *Akkermansia* is a key player in maintaining the gut mucosal barrier and is often associated with favorable metabolic health (79–81). Crucially, the observation that *Akkermansia* enrichment persists even amidst diminished SCFA production may imply that the ketogenic metabolic state, distinct from fiber-dependent fermentation, establishes a unique ecological niche for this immunogenic taxon. Most notably, its presence is a strong predictor of positive response to ICIs, particularly improved anti-PD-1 efficacy (10,76). Within an oncological framework, this targeted microbial shift may function as a robust biological adjuvant, as Akkermansia has been shown to increase the recruitment of highly immunogenic T cells to the tumor microenvironment (10,82) and to induce antigen-specific T cell responses and IgG1 antibodies (83). Since the KD has been observed to enrich specific taxa such as *A. muciniphila*—a bacterium causally linked to improved PD-1 blockade outcomes in mouse models and patient cohorts— there is a strong biological rationale that the diet may prime the gut ecosystem for immunotherapy success (10,84).

At the time of this review, the translation of the microbial shifts from mice and non-cancer cohorts to oncology remains a critical knowledge gap, necessitating a deeper exploration of how KD-driven dysbiosis influences the tumor microenvironment. Collectively, these complex findings have generated interest in the intersection between the gut microbiome and ketogenic diet in cancer patients. Future clinical trials focusing on oncological populations are essential to determine whether the systemic metabolic shifts of ketosis directly foster a more immunogenic gut environment. By analyzing longitudinal microbiome samples alongside treatment responses, researchers can distinguish between the diet’s direct metabolic impacts and those mediated specifically by the gut microbiome. Establishing this link is critical for validating the ketogenic diet as a functional biological adjunct to current immunotherapy protocols.

Among the five trials identified in this review that explicitly incorporate microbiome-related endpoints, all five were launched after 2020, underscoring the recent shift toward integrating microbiome analysis into KD-focused oncology research. Notably, both in-progress studies (NCT06391099 and NCT06896552) plan to investigate the relationship between the gut microbiome, immunotherapy, and cancer. From our knowledge, these will be the first human studies to explore these key interactions. Additionally, the pending results from a separate study (NCT04631445) will provide details on the effects of MSKD on the microbiome in pancreatic cancer patients receiving triplet chemotherapy. Together, these studies represent the early efforts to understand how KD may alter the gut microbiota and impact clinical outcomes in cancer therapies.

### Adherence and Feasibility Among Trials

Based upon our review, 266/378 (70%) of patients were either randomized or enrolled into the KD arm for each study. From our results, we found that adherence was typically measured by some form of ketone measurement, typically BHB concentrations. However, specific methods of measuring ketones varied and included other parameters, such as urinary ketones, insulin levels, and plasma ketone levels. In the context of completed clinical trials, 14/20 (70%) reported explicit adherence measurements. Most commonly, ketosis was defined as ≥0.5 mmol/L BHB levels. Notably, Van der Louw et al. reported basepoint levels as high as >3 mmol/L BHB concentration (85), which would be unusual if the persons were not following a KD, on an SGLT2i, or insulin insufficient. From a nutritional perspective, nutritional ketosis is most defined as circulating ketone levels of >0.5 mmol/L (86). Most common reasons for discontinuation were attributed to dietary burden or failure to comply with the prescribed macronutrient ratios. In the context of dietary forms, eight different forms of ketogenic diet (LCD, MSKD, IID, MCTKD, MKD, MAD, WFKD, KD) were implemented across 20 completed trials. Lower ketogenic diet macronutrient ratios, along with an increase in protein consumption, can often increase dietary adherence (87). It should be noted that the classic 4:1 KD often achieves ketosis faster and may be more difficult to adhere to, while diet forms such as 1.5:1 MAD can lead to better adherence, but may not be considered as effective as KD (87). As a result, many studies from our findings varied in dietary forms, intervention duration, and adherence levels. This heterogeneity and lack of transparency complicate effective cross-trial comparisons, demonstrating a clear need for more consensus on ketogenic diet nomenclature, formulation and implementation approaches, and adherence monitoring.

### Limitations of Current Clinical Evidence and Study Design

Despite the emerging interest in ketogenic diets as a form of cancer therapy, the current clinical evidence base remains constrained by several key factors. RCTs continue to be recognized as the gold standard for evaluating efficacy and potential for new therapies in oncology (88–90). To date, only four RCTs have been completed evaluating KD intervention and cancer, while only three RCTs have fully published their results. In the three RCTs with fully published results, 193 total patients were randomized, with 97 (50%) assigned to the KD intervention. In total, 71 (73%) of KD participants successfully completed their assigned diet until the final endpoint over a mean trial duration of 13.33 weeks. Shorter study windows with fewer time points can make it difficult to estimate the impact of an intervention with precision, unless compensated for with a significant sample size increase (91). Conversely, recruitment and adherence remain a problem in longer-term dietary interventions, which can, in turn, result in a study being statistically underpowered (92). In terms of other trial designs, 16 (80%) trials were identified as single-group, parallel, or crossover assignments. While some non-randomized trials provide valuable insight and data on complex oncological topics, differences in findings may be a result of systemic differences, and treatment outcomes may be overestimated (93).

From our search, we identified eight terminated trials and two withdrawn trials, highlighting relevant issues in KD intervention and oncology trials. From these trials, 8 (80%) indicated issues with maintaining a patient population, accrual, and recruitment. Notably, one trial (NCT05119010) planned to evaluate the objective response rate to the combination of Nivolumab and Ipilimumab administered alongside either a continuous or intermittent ketogenic diet in renal cell carcinoma patients. Additionally, this study aimed to evaluate how ketosis may alter the gut microbiota and impact the response to immunotherapy. However, this study was ultimately terminated due to various recruitment challenges, including competition with another microbiome trial, patient refusal to participate in a dietary intervention, poor tolerance of DPD, and issues with the biospecimen sample collection process. Furthermore, two studies (NCT01419483 and NCT01419587) explore the safety of 4:1 KD intervention with concurrent chemoradiation in pancreatic and lung cancer patients, respectively, and both were terminated in 2017 due to poor accrual. Patients reported difficulty complying with the KD due to its oiliness and restrictive nature, with the study team suggesting that a 3:1 KD or a modified Atkins diet may enhance subject adherence (94). Another study (NCT03075514) exploring the feasibility of KD in glioblastoma (GB) patients was successfully completed but also highlighted challenges throughout their study. Specifically, the study team experienced a lower-than-expected recruitment rate (28.6%) and a low retention rate at the final 3-month endpoint (33%), which led the researchers to recommend a shorter six-week KD intervention aligned with concurrent chemoradiotherapy in GB patients (45). Additionally, the study team made an amendment that included a modified ketogenic diet arm, which allowed more flexibility for patients and was less restrictive when compared to the initial medium-chain triglyceride ketogenic diet (45). These challenges highlight the limitations of the current clinical evidence base and the implementation of rigid ketogenic dietary protocols in oncology settings. Future research must consider the relevance of randomization, population size, dietary length and approach in future studies to ensure successful adherence and enforce scientific rigor and relevance.

### Future Directions for Ketogenic Diet and Oncology Trials

The evolving landscape of ketogenic diet research in oncology highlights several key areas for future investigation that are essential for relevant clinical outcomes. First, given the increasing interest in concurrent immunotherapy treatment with ketogenic diet, in-progress and future trials should consider incorporating more immunotherapy-based primary endpoints, such as overall survival, event-free survival, and pathological response (95). Additionally, future trials evaluating KD as a metabolic adjuvant to ICIs should also consider adding objective response rate, progression-free survival, and assessment of immune-related adverse events as other study outcomes. Similarly, emerging microbiome-focused studies should prioritize longitudinal microbiome profiling to clarify interactions between diet, host metabolism, and microbial communities. Trials should evaluate whether fortified KD protocols incorporating fiber sources can mitigate the observed depletion of SCFA-producing taxa while maintaining therapeutic ketosis. Due to the limited nature of clinical evidence and literature, such endpoints would provide critical information on how KD modifies the microbiome in cancer patients and its influence on treatment response or immune modulation over time. Third, it is imperative that future studies report transparent and accurate results, particularly those related to adherence, ketosis biomarkers, and dietary design. These results are significant in understanding the relevance of metabolic changes, treatment outcomes, and study design for future trials. Due to the complex nature of dietary interventions, future researchers should consider personalized dietary strategies and tailored nutrient approaches (96). One suggestion to accomplish this is the integration of different omics technologies, which can tailor dietary interventions to specific genetic and microbial profiles and stratify populations, leading to better population representation and more precise outcome interpretation (97). These tailored nutritional approaches should also account for tumor type, concurrent therapy, and patient preferences, which include flexibility in diet formulation leading to improved adherence and patient accrual across trials. Fourth, the significance of results from randomized and non-randomized trials should be considered for the design of future studies. RCTs may offer more rigor, providing stronger evidence and statistically powerful interpretations of the relationship between immunotherapy, gut microbiota, and ketogenic diet. Finally, lessons from previously terminated or withdrawn trials should be considered when designing future studies. Overall, these directions represent critical next steps toward optimizing the clinical relevance, feasibility, and therapeutic impact of KD in cancer care, with specific pertinence to immunotherapy and the gut microbiome.

### Conclusions

To our knowledge, this is the first review investigating ketogenic dietary intervention clinical trials in cancer, with a focus on immunotherapy and the gut microbiome. As interest in metabolic therapies for cancer continues to grow, the ketogenic diet has attracted interest as a promising yet underexplored adjunct to immunotherapy, with the gut microbiome serving as a potential mediator of its therapeutic effects. This review explores the rapidly growing clinical trial evidence base, which highlights the challenges and potential of implementing KD in various oncology trials. While pre-clinical studies offer valuable and promising insights into immunomodulatory mechanisms and microbial shifts associated with KD, clinical evidence remains in its early stages, with limited clinical evidence currently available. Current efforts and in-progress trials demonstrate the growing interest in the field. For future studies, well-designed trials that integrate immunotherapy endpoints, longitudinal microbiome analysis, and individualized dietary strategies are crucial to fully understand the therapeutic potential of KD in cancer therapies. By addressing these gaps, future research can elucidate how nutritional modulation can optimally influence the gut microbiota and immune response, ultimately improving outcomes for patients undergoing cancer immunotherapy, as well as other concurrent therapies.

## Figures and Tables

**Table 1.** Completed ketogenic diet intervention and cancer clinical trials overview.

Trial ID	Name	Design	*N* (enrolled)	*N* (completed)	Cancer type	Diet	Length	Concurrent treatment

NCT03171506	Targeted Disruption to Cancer Metabolism Through Dietary Macronutrient Modification	RCT	*n* = 73: *n* = 36 (control), *n* = 37 (KD)	*n* = 45: *n* = 20 (control), *n* = 25 (KD)	Ovarian, Endometrial	KD: 70%:25%:5% [fat:protein:CHO]	12 weeks	Chemotherapy: control (*n* = 4), KD (*n* = 7)
IRCT20171105037259N2	Effect of Ketogenic diet on patients with locally advanced and metastatic breast cancer	RCT	*n* = 80: *n* = 40 (control), *n* = 40 (KD)	*n* = 60: *n* = 30 (control), *n* = 30 (KD)	Breast	KD: 55%:19%:6% [fat:protein:CHO], 20% from MCT	12 weeks	Chemotherapy
NCT03535701	Ketogenic Diet and Chemotherapy to Affect Recurrence of Breast Cancer (The KETO-CARE Study)	Parallel Assignment	*n* = 20	*n* = 17: *n* = 15 (phase 1), *n* = 9 (phase 2)	Breast	WFKD: 20–25 g/day CHO, 1.2–1.5 g protein/kg reference weight, dietary fat to satiety	6 months: 3 months (phase 1), 3 months (phase 2)	Chemotherapy
NCT01716468	A Low-Carbohydrate Diet for Advanced or Metastatic Cancer	Single Group Assignment	*n* = 11	*n* = 6 (8 weeks), *n* = 4 (16 weeks), *n* = 3 (≥16 weeks)	Prostate, Skin, Renal cell, Pancreatic, Colon, Thyroid, Head and Neck, Lung	MAD: 20–40 g/day CHO	4–16 weeks	None
NCT03075514	Ketogenic Diets as an Adjuvant Therapy in Glioblastoma: A Randomized Pilot Trial	Parallel Assignment (randomized)	*n* = 12: *n* = 6 (MCTKD), *n* = 6 (MKD))	*n* = 4 (12 weeks): *n* = 3 (MCTKD), n=1 (MKD). *n* = 3 (12 months): *n* = 2 (MCTKD), n=1 (MKD)	Glioblastoma multiforme	MKD: 80% fat, 5% CHO. MCTKD: 75% fat (30% via MCT), 10% CHO	12 weeks/12 months	Chemoradiation
**N/A**	The Impact of a Ketogenic Dietary Intervention on the Quality of Life of Stage II and III Cancer Patients: A Randomized Controlled Trial in the Caribbean	RCT	*n* = 40: *n* = 20 (control), *n* = 20 (KD)	*n* = 36: *n* = 20 (control), *n* = 16 (KD)	Breast, Prostate, Colon/rectal, Cervical, Lung	MKD: 75%15%:10% [fat:protein:CHO]	16 weeks	Chemotherapy or Radiotherapy
**N/A**	Effects of a ketogenic diet on the quality of life in 16 patients with advanced cancer: A pilot trial	Single Group Assignment	*n* = 16	*n* = 5 (completed intervention), *n* = 7 (included in analysis)	Ovarian, Esophagus, Pancreatic, Thyroid, Colon	KD: <70g/day CHO. Oil protein/fat shake (21 g:14g:5g) (fat:protein:CHO), 2 times daily	12 weeks	None

Overview of retrieved completed KD intervention and cancer studies – Part A. Includes trial design, sample size, cancer type, diet variation, diet length, and concurrent treatment.

**Abbreviations: RCT** – randomized controlled trial; **KD** - ketogenic diet; **CHO** – carbohydrate; **MCT** – medium-chain triglyceride **WFKD** – well-formulated ketogenic diet; **MAD** – modified Atkins diet; **MKD** – modified ketogenic diet; **MCTKD** – medium-chain triglyceride ketogenic diet.

**Table 2.** Completed ketogenic diet intervention and cancer clinical trials overview.

Trial ID	Name	Design	*N* (enrolled)	*N* (completed)	Cancer type	Diet	Length	Concurrent treatment

NCT00444054	Pilot Feasibility Study Of A Low Carbohydrate Diet In Patients With Advanced Cancer	Single Group Assignment	*n* = 12	*n* = 10	Breast, Fallopian tube, Colorectum, Lung, Esophagus, Ovarian	IID: <5% CLIO for energy intake	26–28 days	None
NCT01865162/NCT02302235	Ketogenic Diet as Adjunctive Treatment in Refractory/End-stage Glioblastoma Multiforme: a Pilot Study/Ketogenic Diet Treatment Adjunctive to Radiation and Chemotherapy in Glioblastoma Multiforme: a Pilot Study	Single Group Assignment	*n* = 8: *n* = 4 (newly diagnosed GBM), *n* = 4 (recurrent GBM)	*n* = 5: *n* = 4 (newly diagnosed GBM), *n* = 1 (recurrent GBM)	Glioblastoma multiforme	KD: 4:1 [fat:protein + CHO], lOg/day CLIO	6 months	Chemoradiation
NTR5167	Ketogenic diet treatment as adjuvant to standard treatment of glioblastoma multiforme: a feasibility and safety study	Single Group Assignment	*n* = 9	*n* = 6	Glioblastoma multiforme	KD: 4:1 [fat:protein + CHO] fluid (6 weeks), 1.5–2:1 KD [fat:protein + CHO] w/ 70% MCT supplement (6 weeks)	14 weeks	Chemoradiation
NCT00575146	Ketogenic Diet for Patients With Recurrent Glioblastoma	Single Group Assignment	*n* = 20	*n* = 17	Glioblastoma multiforme	KD: <60g/day CLIO, Highly fermented yogurt (500 mL/day), Plant oils (basic and addition oils)	12–32 weeks	Chemotherapy
NCT02516501	Investigating the Impact of a Ketogenic Diet Intervention During Radiotherapy on Body Composition: A Pilot Trial	Parallel Assignment	*n* = 63: *n* = *n* = 31 (control), *n* = 32 (KD)	*n* = 59: *n* = *n* = 30 (control), *n* = 29 (KD)	Breast	KD: 75–85% fat, <50g/day CHO, 10 g/meal CHO	35 days (median study duration in KD group), 19–47 days (range)	Radiotherapy
NCT02516501	Investigating the Impact of a Ketogenic Diet Intervention During Radiotherapy on Body Composition: A Pilot Trial	Parallel Assignment	*n* = *n*49: *n* = 25 (control), *n* = 24 (KD)	*n* = 41: *n* = 23 (control), *n* = 18 (KD)	Rectal	KD: ≤50g/day CHO	37 days (median study duration in KD group), 31–43 days (range)	Chemoradiation

Overview of retrieved completed KD intervention and cancer studies – Part B. Includes trial design, sample size, cancer type, diet variation, diet length, and concurrent treatment.

**Abbreviations: RCT** – randomized controlled trial; **IID** – insulin-inhibiting diet; **CHO** – carbohydrate; **KD** - ketogenic diet.

**Table 3.** Completed ketogenic diet intervention and cancer clinical trials overview.

Trial ID	Name	Design	*N* (enrolled)	*N* (completed)	Cancer type	Diet	Length	Concurrent treatment

NCT02516501	Investigating the Impact of a Ketogenic Diet Intervention During Radiotherapy on Body Composition: A Pilot Trial	Parallel Assignment	*n* = 32: *n* = 21 (control), *n* = 11 (KD)	*n* = 28: *n* = 21 (control), *n* = 7 (KD)	Flead and neck	KD: <50g/day CHO	39 days (median study duration in KD group), 35–46 days (range)	Chemoradiation
NCT04631445	Randomized Phase II Trial of Two Different Nutritional Approaches for Patients Receiving Treatment for Their Advanced Pancreatic Cancer	RCT	*n* = 32: *n* = 16 (control), *n* = 16 (KD)	N/A	Pancreatic	MSKD: not specified	N/A	Chemotherapy
NCT02286167	The Feasibility and Biologic Effect of a Modified Atkins-based Intermittent Fasting Diet in Patients With Glioblastoma (GBM)	Single Group Assignment	*n* = 25	*n* = 21	Glioma	MAD: < 50 g CHO, 20% of recommended daily caloric intake (5 days); KD: 4:1 [fat:protein + CHO] liquid (KetoCal drink) (2 days, nonconsecutive fasting)	8 weeks	None
NCT03194516	A Ketogenic Diet Pilot Study for Overweight Prostate Cancer Patients on Active Surveillance	Single Group Assignment	*n* = 10	*n* = 10	Prostate	KD: 3:1 [fat:protein + CHO]	8 weeks	None
NCT02092753	Ketogenic Or LOGI Diet In a Breast Cancer Rehabilitation Intervention (KOLIBRI)	Parallel Assignment	*n* = 153: *n* = 30 (control), *n* = 93 (LCD), *n* = 30 (KD)	*n* = 121: *n* = 25 (control), *n* = 76 (LCD), *n* = 20 (KD)	Breast	KD: 75% fat, 1.4g/kg /day body weight protein, 20–30g/day CHO	20 weeks	None
NCT01092247	The Effect of Ketogenic Diet on Malignant Tumors-Recurrence and Progress	Parallel Assignment	*n* = 9: *n* = 4 (control), *n* = 5 (KD)	*n* = 9: *n* = 4 (control), *n* = 5 (KD)	Glioblastoma multiforme, Gliomatosis cerebri	KD: 4:1 [fat:protein + CHO]	2–31 months	Bevacizumab
NCT02964806	The Potential Use of a Ketogenic Diet in Pancreatobiliary Cancer Patients After Pancreatectomy	Crossover Assignment	*n* = 30: *n* = 10 (control), *n* = 20 (KD)	*n* = 19: *n* = 9 (control), *n* = 10 (KD)	Pancreatobiliary	KD: 1.05–1.75:1 [fat:protein + CHO]	~1 month	None

Overview of retrieved completed KD intervention and cancer studies – Part C. Includes trial design, sample size, cancer type, diet variation, diet length, and concurrent treatment.

**Abbreviations: RCT** – randomized controlled trial; **KD** - ketogenic diet; **CHO** – carbohydrate; **MSKD** – medically supervised ketogenic diet; **MAD** – modified Atkins diet; **MKD** – modified ketogenic diet.

**Table 4.** Completed ketogenic diet intervention and cancer clinical trials results and adherence.

Trial ID	Name	Results	Ketosis biomarkers	Adherence definition

NCT03171506	Targeted Disruption to Cancer Metabolism Through Dietary Macronutrient Modification	KD improved physical function, shown by a 4-point increase in SF-12 Physical Component Summary scores. The KD group experienced significantly reduced cravings for starchy foods, sweets, and fast-food fats (*p* = 0.0004 overall). Women not receiving chemotherapy reported a 23% increase in perceived energy (*p* = 0.02).	Associated with urinary ketones	Mean serum BHB concentrations (week 12): control (0.25 ±0.04 mmol/L), KD (0.91 ±0.16 mmol/L), *p* < 0.001. Pts (*n* = 4) withdrew due to inability to comply with diet.
IRCT20171105037259N2	Effect of Ketogenic diet on patients with locally advanced and metastatic breast cancer	The KD group experienced reductions in body weight, BMI, and fat percentage, with positive changes in lipid and liver profiles. OS was improved in neoadjuvant breast cancer patients receiving KD. Reduction in tumor sizes 26mm (KD group), 6 mm (control group), (*p* = 0.01). TNF-α decreased (Median: 0.64 [CI 95%: −3.7, 5], *p* < 0.001), IL-10 increased (MD: 0.95 [CI 95%: −1,3], *p* < 0.001) in the KD group.	>0.3mmol/l serum BHB concentration	89% of KD pts achieved ketosis and were considered adherent to the diet. Pts (*n* = 2) dropped out due to inability to comply with diet.
NCT03535701	Ketogenic Diet and Chemotherapy to Affect Recurrence of Breast Cancer (The KETO-CARE Study)	Fasting glucose decreased in 80% of pts (*n* = 13) after Phase 1. insulin sensitivity improved in 87% of pts (*n* = 13) after 3 months. Higher glucose variability was predictive of dropout. KD mitigated chemotherapy-induced hyperglycemia and insulin resistance. Participants experienced weight loss, primarily from fat mass, not lean mass and reduction of glycolytic demand of diet.	0.5–4.0 mmol/L serum BHB concentration	Capillary BHB: phase 1 (mea *n* = 0.8mmol/l), phase 2 (mea *n* = 0.7mmol/l). Attrition rate: phase 1 (47%) and phase 2 (75%). Participants remained in metabolic state 90% of time after attaining nutritional ketosis. Pts who withdrew were not related to diet.
NCT01716468	A Low-Carbohydrate Diet for Advanced or Metastatic Cancer	MAD was well tolerated in advanced cancer pts, with stable or improved quality of life and no significant adverse effects on renal, metabolic, or hematologic parameters. Among pts who remained on the diet for 16 weeks, especially those with melanoma, several experienced stable diseases and significantly outlived their expected survival times.	0.3–1.0 mmol/L serum BHB concentration	70% (*n* = 7) pts reached ketosis within 2 days into intervention. Pt (*n* = 1) was removed due to only being able to achieve ketosis 2 out of the first 4 weeks. Serum ketone levels mean: week 4 (22.08mg/dl ± 10), week 8 (7.4mg/dl ± 4.81), week 16 (6.17 mg/dl ±7.99). This decrease was not statistically significant (*p* = 0.875), which indicates good adherence and ability to maintain ketosis with time.

Overview of retrieved completed KD intervention and cancer studies – Part A. Includes measured outcomes, ketosis biomarkers, and reported compliance and dietary adherence.

**Abbreviations: KD** - ketogenic diet; **MAD** – modified Atkins diet; **BHB** – β-hydroxybutyrate; **Pts** – patients; **OS** – overall survival rate; **QoL** – quality of life.

**Table 5.** Completed ketogenic diet intervention and cancer clinical trials results and adherence.

Trial ID	Name	Results	Ketosis biomarkers	Adherence definition

NCT04631445	Randomized Phase II Trial of Two Different Nutritional Approaches for Patients Receiving Treatment for Their Advanced Pancreatic Cancer	Initial results: MSKD with triplet chemotherapy was feasible and safe. No significant differences between groups in insulin, HbAlc, or weight change (*p* > 0.05), and adverse (Gr 1–2 fatigue, constipation, weight loss) events were mild.	0.5–3.0mmol/L BHB levels	KD pts (*n* = 15) achieved nutritional ketosis. Mean KD BHB: 0.57mmol/L (95% CI 0.40–0.73). Median proportion days in ketosis: 39.4% (range 0–95.8%).
NCT03194516	A Ketogenic Diet Pilot Study for Overweight Prostate Cancer Patients on Active Surveillance	KD led to weight and BMI reductions (7.4%, *p* = 0.0003). PSA and inflammatory markers (CRP and IL-6) remained stable (*p* > 0.05). Half of pts showed tumor remission or downgrading on re-biopsy, while only two exhibited progressions. Short-term KD is a feasible and potentially beneficial intervention for weight loss without worsening inflammation in men with low-risk prostate cancer.	>0.3mmol/L ketone levels	Mean blood ketone levels (end of KD intervention): 0.32 (0.12) mmol/L. Pt (*n* = 1) did not achieve 0.3mmol/L, but completed the intervention and maintained ketone levels 0.2–0.27 mmol/L.
NCT02092753	Ketogenic Or LOGI Diet In a Breast Cancer Rehabilitation Intervention (KOLIBRI)	KD pts had lower BMI (*p* = 0.0003), fat mass (*p* = 0.0002), and visceral fat (*p* = 0.0002), but higher metastatic burden (*p* = 0.0006) vs. LCD at baseline. KD pts had higher energy intake and maintained muscle mass and ketosis. KD showed improved body composition, QoL, and TG/HDL ratio (0.9). IGF-1 showed no substantial change. KD is feasible and metabolically beneficial for breast cancer pts.	Associated with daily urine ketone tests	KD pts reached the intended ketogenic ratio of 1.6:1 (mean: 1.65 ±0.08) and stable ketosis was exhibited.
NCT01092247	The Effect of Ketogenic Diet on Malignant Tumors-Recurrence and Progress	Single pt with gliomatosis cerebri received KD as monotherapy and experienced the best metabolic and radiological response (stable disease over 31 months, with sustained urine ketosis and brain metabolite changes). Radiologic improvements could not be solely attributed to KD due to concurrent bevacizumab treatment.	>2 ketone level	1H-MRS monitored metabolic changes in KD pts, detecting brain ketone bodies (acetone and acetoacetate) in only (*n* = 2) KD pts, despite high urine ketone levels in (*n* = 4) KD pts. Urine ketosis was high (>4) in pts who maintained diet.
NCT02964806	The Potential Use of a Ketogenic Diet in Pancreatobiliary Cancer Patients After Pancreatectomy	Post-pancreatectomy cancer pts (KD group) had improved meal satisfaction, energy intake, and body composition, without increased digestive complications. Body fat mass decreased significantly in the KD group (*p* < 0.05), and lean body mass was better preserved. KD induced systemic metabolic changes (elevated ketones) and provided preliminary evidence of nutritional and metabolic benefits.	Association not established	Ketone detection frequency (KD): 22.2±23.7%, (Control): 50.8±35.1%, (*p* = 0.065). KD group had significantly higher serum ketone body concentration (BHB). Increase in urine ketone bodies (~2 week average). KD group had higher adherence than control. Pts (*n* = 6) dropped out due to refusal to eat KD.

Overview of retrieved completed KD intervention and cancer studies – Part B. Includes measured outcomes, ketosis biomarkers, and reported compliance and dietary adherence.

**Abbreviations: MSKD** – medically supervised ketogenic diet; **KD** - ketogenic diet; **BHB** – β-hydroxybutyrate; **Pts** – patients; **LCD** – low carbohydrate diet; **QoL** – quality of life; **IGF-1** – insulin-like growth factor 1.

**Table 6.** Completed ketogenic diet intervention and cancer clinical trials results and adherence.

Trial ID	Name	Results	Ketosis biomarkers	Adherence definition

NCT02516501	Investigating the Impact of a Ketogenic Diet Intervention During Radiotherapy on Body Composition: A Pilot Trial	Pts on KD in combination with chemoradiation therapy experienced significant reductions in fat mass (−2.8 kg, *p*<0.0001) and body weight (−4.1 kg, *p* = 0.0005), while preserving skeletal muscle mass. Fat loss occurred without significant muscle wasting, suggesting potential anti-tumor benefits. In an intention-to-treat analysis, the KD group showed significantly higher rates of near-complete tumor response (43% vs. 15%, *p* = 0.018) and higher mean Dworak tumor regression grades (2.4 vs. 1.8, *p* = 0.023).	≥0.5 mmol/L BHB levels	Median BHB (KD group): 0.7mmol/l, range: (0.2–3.2 mmol/l). *n* = 7 pts achieved >0.5 mmol/l BHB measurements All KD pts achieved at least one >0.4 mmol/l BHB measurement. 4 pts dropped out KD group (*n* = 2 due to non-adherence with KD).
NCT02516501	Investigating the Impact of a Ketogenic Diet Intervention During Radiotherapy on Body Composition: A Pilot Trial	KD was associated with nonsignificant but favorable trends, including a weekly increase in skeletal muscle mass (+0.17 ± 0.08 kg, *p* = 0.060) in pts. The control group showed significant reductions in all body composition parameters. No statistically significant differences were noted in PFS or OS between KD and SD groups. KD median OS and PFS follow-up: 35.2 months (range: 12.4–63.7 months) and 35.2 months (range: 4.3–63.7 months). Control median OS and PFS follow-up: 45.8 months (range: 6.7–78.0 months) and 36.9 months (range: 6.7–70.4 months).	≥0.5 mmol/L BHB levels	Median BHB (KD group): 0.7 mmol/l, range: (0.2–3.2 mmol/l). *n* = 7 pts achieved ≥0.5 mmol/l BHB measurements All KD pts achieved at least one ≥0.4 mmol/l BHB measurement. 4 pts dropped out KD group (*n* = 2 due to non-adherence with KD).
NCT02286167	The Feasibility and Biologic Effect of a Modified Atkins-based Intermittent Fasting Diet in Patients With Glioblastoma (GBM)	Intervention significantly lowered systemic markers of glucose metabolism such as hemoglobin Ale and insulin but did not affect fasting glucose or IGF-1 levels. Cerebral metabolic changes included a decrease in tumor-region phosphocholine and altered glutamine metabolism. Weight and BMI decreased slightly, but fat-free mass increased, which indicates preserved nutritional status.	Associated with average of home post fast and post-MAD day ketone measures (weeks 2–8) (average ketonuria)	48% (*n* = 12) of pts were adherent with the intervention. 72% (*n* = 18) of pts were adherent with MAD and fasting interventions (but had one day of ≥40 g CHO). Sustained systemic and cerebral ketosis was induced (increased urine ketones (AcAc) and cerebral ketone bodies (β-hydroxybutyrate and acetone)). 80% of participants achieved moderate or greater ketonuria, which moderately correlated with cerebral ketone levels.

Overview of retrieved completed KD intervention and cancer studies – Part C. Includes measured outcomes, ketosis biomarkers, and reported compliance and dietary adherence.

**Abbreviations: KD** – ketogenic diet; **MAD** – modified Atkins diet; **BHB** – β-hydroxybutyrate; **Pts** – patients; **OS** – overall survival rate; **PFS**- progression-free survival; **IGF-1** – insulin-like growth factor 1.

**Table 7.** Completed ketogenic diet intervention and cancer clinical trials results and adherence.

Trial ID	Name	Results	Ketosis biomarkers	Adherence definition

NCT03075514	Ketogenic Diets as an Adjuvant Therapy in Glioblastoma: A Randomized Pilot Trial	GHS declined below brain cancer reference levels by week six for those who withdrew (MCTKD: 41.7, MKD: 50), but improved or remained stable for those who remained, most notable in the MKD group (MKD: 100). Median PFS was 14.4 weeks and OS was 67.3 weeks. Some pts reported improved sense of control and MRI scans.	≥0.4 mmol/l serum and urinary ketones	79.7% (*n* = 3) MCTKD pts and 79.3% (*n* = 3) MKD pts recorded >4 mmol/l within the first 6 weeks. Pts who withdrew had lower urinary and serum ketone levels. MCTKD (*n* = 1) pt, MKF (*n* = 2) pts withdrew due to dietary burden. Food acceptability baseline: MCTKD 60.7 ±10.5 (*n* = 6), MKD 54.3 ±6.2 (*n* = 6). endpoint: MCTKD 47.5 ±6.5 (*n* = 2); MKD 53 (*n* = 1).
**N/A**	The Impact of a Ketogenic Dietary Intervention on the Quality of Life of Stage II and III Cancer Patients: A Randomized Controlled Trial in the Caribbean	MKD pts experienced metabolic and psychosocial benefits, including reductions in weight (−8.55 kg), BMI (−1.32), fasting blood glucose (−11.35 mg/dL), cholesterol (−12.75 mg/dL), systolic blood pressure (−6.8 mmHg), and improvements in QoL (+28 points) and patient health scores (+10 points). The MKD group demonstrated improvements in mental health and psychosocial well-being over time.	>0.5 mmol/L urinary ketones	Majority of patients took ~2 weeks to achieve and maintain ketosis.
**N/A**	Effects of a ketogenic diet on the quality of life in 16 patients with advanced cancer: A pilot trial	Emotional functioning and insomnia improved despite overall disease progression. Beneficial metabolic changes were observed, including reduced LDL (from 108 ± 36 to 92 ± 45 mg/dL; *p* < 0.01), lowered ALT (from 29.9 ± 22.2 to 25.9 ± 11.9 U/L; *p* < 0.01), and increased leukocyte counts (from 5.5 ± 1.5 to 6.4 ± 1.2 × 10^3^/pL; *p* < 0.001).	≥0.5 mmol/L ketone bodies	Pts (*n* = 3) dropped out due to inability to adhere to diet. 60% of patients who completed diet were considered adherent by ketosis biomarkers. Values varied between 0.5 and 8 mmol/l (predominantly 1.5–4.0 mM). Evidence of stable ketosis in pts (*n* = 3) but dropped out due to progression by weeks 6–8.
NCT00444054	Pilot Feasibility Study Of A Low Carbohydrate Diet In Patients With Advanced Cancer	Pts with stable disease or partial remission had significantly higher metabolic response (mean SEM: 16.6± 3.2) than those with progressive disease (mean SEM: 5.2 ± 1.9), suggesting a possible link between greater ketosis and tumor control. Mean caloric deficit (35%) and weight loss (4%) across participants did not correlate with outcomes, which indicates ketosis is more strongly associated with tumor response than calorie restriction.	Associated inversely with insulin serum levels (P = 0.03)	Mean BHB at protocol/baseline (relative ketosis): Mean ± SEM, (10.9 ± 1.7, P < 0.01)

Overview of retrieved completed KD intervention and cancer studies – Part D. Includes measured outcomes, ketosis biomarkers, and reported compliance and dietary adherence.

**Abbreviations: MCTKD** – medium-chain triglyceride ketogenic diet; **MKD** – modified ketogenic diet; **KD** - ketogenic diet; **BHB** – β-hydroxybutyrate; **Pts** – patients; **OS** – overall survival rate; **PFS**- progression-free survival; **GHS** – global health status; **QoL** – quality of life.

**Table 8.** Completed ketogenic diet intervention and cancer clinical trials results and adherence.

Trial ID	Name	Results	Ketosis biomarkers	Adherence definition

NCT01865162/NCT02302235	Ketogenic Diet as Adjunctive Treatment in Refractory/End-stage Glioblastoma Multiforme: a Pilot Study/ Ketogenic Diet Treatment Adjunctive to Radiation and Chemotherapy in Glioblastoma Multiforme: a Pilot Study	KD is feasible and well tolerated over a 6-month period in glioblastoma pts. 4/7 patients had full adherence and pts (*n* = 4) continued the diet voluntarily beyond the study, up to 26 months. Small sample size provides limitations in making conclusion about KD efficacy.	Associated with urine/blood ketone diaries, monthly serum BHB levels	Pt adherence was graded upon the following scale: 0–3 (0= none, 1 = partial-slight, 2 = partial-substantial, 3 = complete). Results: adherence level 3 (*n* = 3), adherence level 2 (*n* = 2), adherence level 1 (*n* = 0), adherence level 0 (*n* = 1), adherence level N/A (*n* = 1).
NTR5167	Ketogenic diet treatment as adjuvant to standard treatment of glioblastoma multiforme: a feasibility and safety study	KD and chemoradiation in glioblastoma (GBM) is feasible and safe. However, pts required dietician support to maintain adherence. QoL measures showed minor declines in areas like global QoL, fatigue, and insomnia. The median OS was 12.8 months. All pts continued a carbohydrate-restricted diet, with some pts (*n* = 2) experiencing prolonged survival.	≥3 mmol/L blood BHB concentration	67% (*n* = 6) pts were adherent and adhered to the diet for 14 weeks. Pts reached ketosis (*n* = 9) in a mean of 4.5 days. Mean ketone level: 4.3 mmol/L (first 6 weeks), 2.9 mmol/L (last 6 weeks).
NCT00575146	Ketogenic Diet for Patients With Recurrent Glioblastoma	No serious diet-related adverse events, only weak, transient symptoms (mild hunger or sugar cravings) were reported. PFS on KD was limited (5 weeks), while pts who achieved stable ketosis (*n* = 8) 6 weeks had a non-significant trend toward longer PFS, than no stable ketosis (*n* = 5) pts, (*p* = 0.069). Pts who continued the diet while receiving salvage therapy with bevacizumab (response rate 85%), had a higher median PFS (20.1 weeks), than what was seen in a matched non-diet cohort (16.1 weeks).	>0.5 mmol/l urinary ketones	Fraction ketone positive measurements / all measurements reported in pts (*n* = 13) (0, 0.03, 0.3, 0.43, 0.47, 0,67, 0,94, 0,95, 0,96, 0,97, 0.99, 1.0, 1.0).
NCT02516501	Investigating the Impact of a Ketogenic Diet Intervention During Radiotherapy on Body Composition: A Pilot Trial	KD during radiotherapy is feasible and well-tolerated (9% dropout rate), with no significant adverse events. KD pts experienced significant weekly reductions in body weight and fat mass (0.4 kg/week each), with greater total losses in body weight (2.9± 2.2 kg) and fat mass (2.3 ± 1.7 kg). KD was associated with metabolic shifts such as decreased T3 hormone levels (0.06 pg/ml/week) and trends toward reduced insulin and IGF-1.	≥0.5 mmol/L BHB levels	Mean/median BHB during radiotherapy: (0.72, 0.49 mmol/L). Median BHB during random point: (0.9 mmol/L, range: 0.3–1.9 mmol/L). Three dropouts during study due to adherence with KD prescription (*n* = 2 pts failed to reach ketosis biomarker).

Overview of retrieved completed KD intervention and cancer studies – Part E. Includes measured outcomes, ketosis biomarkers, and reported compliance and dietary adherence.

**Abbreviations: KD** – ketogenic diet; **BHB** – β-hydroxybutyrate; **Pts** – patients; **OS** – overall survival rate; **PFS**- progression-free survival; **IGF-1** – insulin-like growth factor 1; **QoL** – quality of life.

**Table 9.** In-progress ketogenic diet intervention and cancer clinical trials.

Trial ID	Name	Design	*N*	Cancer type	Diet	Length	Treatment	Purpose

NCT06391099	Ketogenic Dietary Intervention to Improve Response to Immunotherapy in pts With Metastatic Melanoma and Metastatic Kidney Cancer	RCT	*n* = 40: *n* = 20 MM (*n* = 10 KD, *n* = 10 control), *n* = 20 mRCC (*n* = 10 KD, *n* = 10 control)	Renal cell carcinoma, Melanoma	KD: 2:1 [fat:protein + CHO], <50 g/day CHO	24 weeks	Immunotherapy	Evaluate the safety and feasibility of implementing a ketogenic dietary intervention with longitudinal biospecimen collection in oncology clinics, while exploring whether microbiome diversity mediates the relationship between sustained ketosis and tumor response.
NCT06896552	Single-Center Trial on Ketogenic Diet and Immunotherapy in Advanced Cancer This Study Evaluates the Safety and Effects of a Ketogenic Diet (KD) Combined With Immunotherapy in Adults With Advanced Melanoma, cSCC, or RCC	Sequential Assignment (non-randomized)	*n* = 60: *n* = 30 (control), *n* = 30 (KD)	Melanoma, Cutaneous squamous cell carcinoma, Renal cell carcinoma	KD: 60–70%:20–30%:5–10% [fat:protein:CHO], w/supplemental MCT, (2 weeks on, 1 week off)	10 weeks	Immunotherapy	Examine if KD is well-tolerated in cancer pts and if KD improves immune response and treatment effectiveness.
NCT04316520	Ketogenic Diet for Patients Receiving Treatment for Metastatic Renal Cell Carcinoma (CETOREIN)	Single Group Assignment	*n* = 20	Renal cell carcinoma	KD: 2:1 [fat:protein + CHO], w/Betaquik supplement	1 year	Immunotherapy	Examine the efficacy and tolerance of KD with vitamin supplementation.
NCT01535911	Pilot Study of a Metabolic Nutritional Therapy for the Management of Primary Brain Tumors (Ketones)	Single Group Assignment	*n* = 16	Glioblastoma multi forme	KD: 20–25 kcal/day/kg body weight	6 weeks	Chemoradiation	Evaluate if energy restricted KD will decrease or inhibit tumor growth in pts with primary brain cancer.

Overview of retrieved in-progress KD intervention and cancer studies – Part A. Includes trial design, sample size, cancer type, diet variation, diet length, concurrent treatment, and intended study purpose.

**Abbreviations: RCT** – randomized controlled trial; **KD** - ketogenic diet; **CHO** – carbohydrate.

**Table 10.** In-progress ketogenic diet intervention and cancer clinical trials.

Trial ID	Name	Design	*N*	Cancer type	Diet	Length	Treatment	Purpose

NCT05234502	Effects of Ketogenic Diet in Overweight and Obese Women With Breast Cancer	RCT	*n* = 56	Breast	KD: 75%:19%:6% [fat:protein:CHO]	12 weeks	Chemotherapy	Evaluate the effects of a ketogenic diet (KD) compared to a standard healthy diet on chemotherapy-induced sensory and motor neuropathy, tumor response, and overall prognosis in overweight or obese women with breast cancer undergoing neoadjuvant chemotherapy.
NCT05938322	Ketogenic Diet Adherence in Patients Affected llby Locally Advanced Rectal Cancer Patients Who Undergo to Radiotherapy (KOMPARC)	RCT	*n* = 194	Rectal	KD: < 30g g/day CHO, 1.2g–1.5g/day/kg body weight protein/kg, > 65% fat	2 months	Chemoradiation	Examine the effects of KD and adherence in pts with locally Advanced Rectal Cancer pts undergoing chemoradiation therapy.
NCT03451799	Ketogenic Diet in Combination With Standard-of-care Radiation and Temozolomide for Patients With Glioblastoma	Single Group Assignment	*n* = 21	Glioblastoma multiforme	N/A	16 weeks	Chemoradiation	Evaluate the feasibility, safety, tumor response, and impact of a personalized ketogenic diet combined with radiation and temozolomide in pts with glioblastoma.
NCT05708352	A Phase 2 Study of the Ketogenic Diet vs. Standard Anti-cancer Diet Guidance for Patients With Glioblastoma in Combination With Standard-of-care Treatment	RCT	*n* = 170	Glioblastoma multiforme	N/A	18 weeks	Chemo and/or radiationtherapy	Investigates if KD, compared to a standard anti-cancer diet, can improve overall survival in pts with newly diagnosed glioblastoma receiving standard-of-care treatment, while also assessing quality of life, cognitive function, physical activity, and treatment adherence.

Overview of retrieved in-progress KD intervention and cancer studies – Part B. Includes trial design, sample size, cancer type, diet variation, diet length, concurrent treatment, and intended study purpose.

**Abbreviations: RCT** – randomized controlled trial; **KD** - ketogenic diet; **CHO** – carbohydrate.

**Table 11.** In-progress ketogenic diet intervention and cancer clinical trials.

Trial ID	Name	Design	*N*	Cancer type	Diet	Length	Treatment	Purpose

NCT05428852	Keto-Brain:lnvestigating the Use of Ketogenic Diets in Brain Metastases	RCT	*n* = 24: *n* = 12 (control), *n* = 12 (KD)	Brain Metastases	KD: 70–75%:15–20%:<50g CHO [fat:protein:CHO]	16 weeks	Stereotactic radiosurgery	Evaluate if KD is effective in improving treatment response, metabolic outcomes, and quality of life for pts with brain metastases undergoing radiosurgery.
NCT05090358	Preventing High Blood Sugar in People Being Treated for Metastatic Breast Cancer	RCT	*n* = 15	Breast	KD: not specified, or LCD: not specified	12 weeks	(SOC endocrine therapy and SGLT2i Therapy) or PI3K inhibition	Determine if KD, LCD, or canagliflozin can help manage high blood sugar and improve the effectiveness of cancer treatment in pts with metastatic, hormone-receptor positive, PIK3CA-mutant breast cancer who are receiving alpelisib and fulvestrant.
NCT06106139	Ketogenic Diet Improves Thrombocytopenia in Cancer Patients	RCT	*n* = 80	Cancer pts (w/malignant solid tumors)	KD: strict, Circulating: (7 days strict KD, 7 days normal diet), Autonomous: (90%:10%:10%, [fat:protein:CHO]	3 months	Chemoradiation	Evaluate if KD can improve thrombocytopenia (related to chemotherapy) in cancer pts.
NCT05564949	A Ketogenic Diet as a Complementary Treatment on Patients With High-grade Gliomas and Brain Metastases	Single Group Assignment	*n* = 15	Glioblastoma multi forme, Secondary metastases progression	KD: 4:1 [fat:protein + CHO]	3 months	None	Evaluate if a classic KD can extend survival and improve quality of life in pts with high-grade gliomas and brain metastases.
NCT03285152	A Study of Ketogenic Diet in Newly Diagnosed Overweight or Obese Endometrial Cancer Patients	RCT	*n* = 19	Endometrial	KD: 3:1 [fat:protein + CHO]	4 weeks	None	Evaluate the safety, tolerability, and metabolic effects of a KD in newly diagnosed overweight or obese endometrial cancer pts during the presurgical period.

Overview of retrieved in-progress KD intervention and cancer studies – Part C. Includes trial design, sample size, cancer type, diet variation, diet length, concurrent treatment, and intended study purpose.

**Abbreviations: RCT** – randomized controlled trial; **KD** - ketogenic diet; **CHO** – carbohydrate; **LCD** – low carbohydrate diet.

**Table 12.** Terminated or withdrawn ketogenic diet intervention and cancer clinical trials overview.

Trial ID	Status	Name	Design	*N*	Cancer type	Diet	Length	Treatment	Purpose

NCT05119010	Terminated (Recruitment challenges due to competition with another microbiome trial, pt refusal, and poor tolerance of oral DPD)	A Pilot Study Evaluating a Ketogenic Diet Concomitant to Nivolumab and Ipilimumab in pts With Metastatic Renal Cell Carcinoma (KETOREIN)	Parallel Assignment	*n* = 3	Renal cell carcinoma	KD: <40g/day CHO, w/oral liquid ketone supplement BHB monoester (2 tablespoons three times per day for third arm)	1 year	Immunotherapy	Evaluate the objective response rate to the combination of Nivolumab and Ipilimumab administered alongside either KD (continuous or intermittent) or a standard diet, with or without BHB supplementation.
NCT01419483	Terminated (low accrual)	Ketogenic Diet With Concurrent Chemoradiation for Pancreatic Cancer (KETOPAN)	Single Group Assignment	*n* = 5	Pancreatic	KD: 4:1 [fat:protein + CHO]	5 weeks	Chemoradiation	Examine the safety and tolerability of KD combined with chemotherapy and radiation therapy in pts with pancreatic cancer.
NCT00932672	Terminated (slow recruitment, lack of funding, transferred PI)	Atkins Diet and Prostate Cancer Clinical Trial	RCT	*n* = 45	Prostate	AD: <20g/day CHO	6 months	Androgen deprivation therapy	Test the hypothesis that low carbohydrate diet will minimize metabolic consequences of androgen deprivation therapy.
NCT03328858	Terminated (PI unavailable and pts lost to follow up)	Ketogenic Diet in Children With Malignant or Recurrent/Refractory Brain Tumor	Single Group Assignment	*n* = 20	Brain tumor (medulloblastoma, high-grade glioma, low-grade glioma, and ependymoma)	KD: 4:1 [fat:protein + CHO]	1 year	Chemoradiation	Evaluate the effects of KD on QoL and tumor size in pts (pediatric) with malignant or recurrent/refractory brain tumors.

Overview of retrieved terminated or withdrawn KD intervention and cancer studies – Part A. Includes reason for termination or withdraw, trial design, sample size, cancer type, diet variation, diet length, con-current treatment, intended study purpose.

**Abbreviations: RCT** – randomized controlled trial; **Pts** – patients; **CHO** – carbohydrate; **KD** - ketogenic diet; **AD** – Atkins diet; **BHB** – β-hydroxybutyrate.

**Table 13.** Terminated or withdrawn ketogenic diet intervention and cancer clinical trials overview.

Trial ID	Status	Name	Design	*N*	Cancer type	Diet	Length	Treatment	Purpose

NCT04231734	Withdrawn (low accrual)	Ketogenic Diet in Patients With Untreated Low Tumor Burden Mantle Cell Lymphoma	Single Group Assignment	*n* = 0	Mantle cell lymphoma	N/A	12 weeks	N/A	To assess the feasibility and adherence to a KD in pts with low tumor burden, treatment-naive mantle cell lymphoma, while monitoring metabolic, tumor, and body composition changes.
NCT03955068	Withdrawn (lack of funding)	Strict Classic Ketogenic Diet as a Therapy for Recurrent or Progressive and Refractory Brain Tumors in Children	Single Group Assignment	*n* = 0	Brain tumor (regressive or refectory)	KD: 2:1 to 4:1 [fat:protein + CHO] (adjusted based upon BHB levels and adherence)	28 days	N/A	To determine the feasibility of KD of classic, strict KD in pediatric pts with recurrent or progressive and refectory brain tumors and evaluate survival progression and tumor response.
NCT01419587	Terminated (poor accrual and pt adherence)	Ketogenic Diet With Chemoradiation for Lung Cancer (KETOLUNG)	Single Group Assignment	*n* = 5	Non-small lung cancer	KD: 4:1 [fat:protein + CHO]	6 weeks	Chemoradiation	Investigate if KD is tolerable and safe when combine with chemotherapy and radiation therapy in pts with lung cancer.

Overview of retrieved terminated or withdrawn KD intervention and cancer studies – Part B. Includes reason for termination or withdraw, trial design, sample size, cancer type, diet variation, diet length, concurrent treatment, intended study purpose.

**Abbreviations: RCT** – randomized controlled trial; **Pts** – patients; **CHO** – carbohydrate; **KD** – ketogenic diet.

**Table 14.** Terminated or withdrawn ketogenic diet intervention and cancer clinical trials overview.

Trial ID	Status	Name	Design	N	Cancer type	Diet	Length	Treatment	Purpose

NCT02046187	Terminated (excessive protocol deviations due to strict nature of diet requirements	Ketogenic Diet With Radiation and Chemotherapy for Newly Diagnosed Glioblastoma	Single Group Assignment	*n* = 14	Glioblastoma multi forme	KD: 4:1 [fat:protein + CHO] (on chemoradiation), MAD: (on chemotherapy)	8 weeks	Chemoradiation	Evaluates if KD started after surgical resection can be safely maintained and enhance the effectiveness of standard chemoradiotherapy in pts with glioblastoma, and to assess survival, recurrence, and quality of life.
NCT01975766	Terminated (low accrual)	Ketogenic Diet Phase 1 for Head & Neck Cancer	Single Group Assignment	*n* = 14	Squamous cell carcinoma	KD: 4:1 [fat:protein + CHO]	5 weeks	Chemoradiation	Determine whether a very low carbohydrate (ketogenic) diet is safe and tolerable for pts undergoing concurrent chemotherapy and radiation therapy for head and neck cancer.
NCT03785808	Terminated (low accrual)	Reducing Insulin, Growth Hormones, and Tumors (RIGHT)	RCT	*n* = 3	Non-small cell adenocarcinoma, squamous cell carcinoma	KD: 65% fats, starchy vegetables, fruits, berries, and legumes (<10% caloric intake)	24 weeks	N/A	Compare the effects of a low-carbohydrate, high-fat ketogenic diet and a low-fat, high-carbohydrate plant-based diet on biomarkers of inflammation, insulin resistance, and cancer progression in pts with advanced lung cancer.

Overview of retrieved terminated or withdrawn KD intervention and cancer studies – Part C. Includes reason for termination or withdraw, trial design, sample size, cancer type, diet variation, diet length, concurrent treatment, intended study purpose.

**Abbreviations: RCT** – randomized controlled trial; **Pts** – patients; **CHO** – carbohydrate; **KD** - ketogenic diet; **MAD** – modified Atkins diet.

## Data Availability

The data that support the findings of this study are openly available in “How the Ketogenic Diet Shapes the Microbiome to Influence Cancer Immunotherapy Outcomes” at https://doi.org/10.5281/zenodo.17238355.

## Box 1. Description of the common terminology used to describe KD interventions.

Though there are no strict definitions for subtypes of KDs, the following abbreviations are common and reflect different concepts for the ideal formulations of the KD. their typical use is defined here.

MAD = Modified Atkins Diet, less restrictive form of KD, typically 10–20 g/day carbohydrates and 20–30% of energy from protein.

AD = Atkins Diet, low carbohydrate diet with higher protein consumption, often characterized with increasing carbohydrate allowance through phases.

MSKD = Medically Supervised Ketogenic Diet, any form of ketogenic diet that is prescribed, managed, and closely monitored by healthcare professionals.

MKD = Modified Ketogenic Diet, less restrictive variations (e.g., 1:1, 2:1) of classical KD that typically allow higher protein and carbohydrate consumption, often more structured than MAD.

IID = Insulin-Inhibiting Diet, term used to describe low carbohydrate diets that minimize foods that stimulate a large insulin response, emphasizes overall metabolic goal rather than a specific macronutrient ratio.

WFKD = Whole Foods Ketogenic Diet, formulation of ketogenic diet that emphasizes consumption of minimally processed foods and consumption of whole foods, focus on improved nutritional quality while following KD regimen.

## Abbreviations:

(m)RCC: (metastatic) renal cell carcinoma
(m)M: (metastatic) melanoma
ICI: immune checkpoint inhibitors
LCD: low-carbohydrate diet
MSKD: medically supervised ketogenic diet
MCTKD: medium-chain triglyceride ketogenic diet
MKD: modified ketogenic diet
MAD: modified Atkins diet
KD: ketogenic diet
WFKD: well-formulated ketogenic diet
AD: Atkins diet
IID: insulin-inhibiting diet
BHB: β-Hydroxybutyrate
OS: overall survival rate
PFS: progression-free survival
QoL: quality of life
RCT: randomized controlled trial
16S: amplicon sequencing of the 16-Svedberg subunit of the bacterial ribosomal RNA gene
metaG: metagenomic sequencing (shotgun)
